# Multiple infarcted regenerative nodules in liver cirrhosis after decompensation of cirrhosis: a case series

**DOI:** 10.1186/1752-1947-4-375

**Published:** 2010-11-23

**Authors:** Dieter Scholtze, Tanja Reineke, Beat Müllhaupt, Christoph Gubler

**Affiliations:** 1 Department of Internal Medicine, University Hospital Zurich, Raemistrasse 100, 8051 Zurich, Switzerland; 2 Department of Pathology, University Hospital Zurich, Raemistrasse 100, 8051 Zurich, Switzerland; 3 Department of Gastroenterology and Hepatology, University Hospital Zurich, Raemistrasse 100, 8051 Zurich, Switzerland

## Abstract

**Introduction:**

Liver cirrhosis is a common disease with many known complications. Cirrhosis represents a clinical spectrum, ranging from asymptomatic liver disease to hepatic decompensation. Manifestations of hepatic decompensation include variceal bleeding, ascites, hepatic encephalopathy, hepatorenal syndrome, hepatopulmonary syndrome, portopulmonary hypertension and hepatocellular carcinoma. There are reports about infarcted regenerative nodules in cirrhotic livers after gastrointestinal hemorrhage.

**Case presentation:**

We report three Caucasian patients (one female and two male patients; ages: 52, 54 and 60 years) with decompensated liver cirrhosis, who showed newly infarcted regenerative nodules at necropsy. Two of them suffered from gastric variceal bleeding. Histopathology showed extensive infarction in all three cases.

Hemorrhage and inflammatory changes were also observed around the infarcted regenerative nodules.

**Conclusion:**

These patients showed focal liver lesions, to be considered in the differential diagnosis of cirrhotic livers. Infarcted regenerative nodules may be underdiagnosed in patients with decompensation of cirrhosis. In order to differentiate these lesions from malignant tumors, serial imaging seems to be helpful. However, the main differential diagnosis should be an abscess. It is important to know the wide spectrum of image appearances of these lesions. Hypotension can lead to a reduction of portal and arterial liver flow. Since variceal bleeding or septic shock can induce hypotension - as observed in our patients - we conclude that this leads to infarction of such nodules.

## Introduction

Liver cirrhosis is a common disease. Patients with this disease frequently need to be hospitalised due to complications such as variceal bleeding, ascites, hepatic encephalopathy, hepatorenal syndrome, hepatopulmonary syndrome, portopulmonary hypertension or hepatocellular cancer. There are some reports about infarcted regenerative nodules in liver cirrhosis in patients with gastric variceal bleeding [1,2]. Recently, we observed three cases with infarction of regenerative nodules in Caucasian patients with decompensated liver cirrhosis.

## Case presentation

During the course of a year, 141 patients with liver cirrhosis were hospitalised. Forty-three patients presented as Child-Pugh A, 48 as Child-Pugh B and 50 as Child-Pugh C. The reason for the hospitalisation was most frequently a decompensation. Decompensation was defined as ascites (n = 39, encephalopathy (n = 22), gastric variceal bleeding (n = 48) and/or others, for example, liver transplantation, chemoembolisation of a hepatocellular carcinoma (n = 32). Decompensation was most prominent (n = 48) in patients with Child C cirrhosis. Overall, 91 (65%) patients were suffering from decompensated liver cirrhosis. The reason for hospitalisation in Child A cirrhosis was a newly detected hepatocellular carcinoma.

In 12 (8.5%) patients contrast-enhanced computed tomography (CT) (n = 9) scans or magnetic resonance imaging (MRI) (n = 3) showed advanced liver cirrhosis with regenerative nodules, in 11 (7.8%) patients a hepatocellular carcinoma was observed. The definition of a regenerative nodule is a non-cirrhotic non-neoplastic nodular transformation of the liver parenchyma. It consists of nodular regenerative hyperplasia thought to be caused by portal venous thrombosis, the closely related partial nodular transformation which is limited to the perihilar region near the porta hepatis and focal nodular hyperplasia due to arterial hyperplasia with nodular parenchymal hyperplasia and cholestasis.

**Figure 1:**
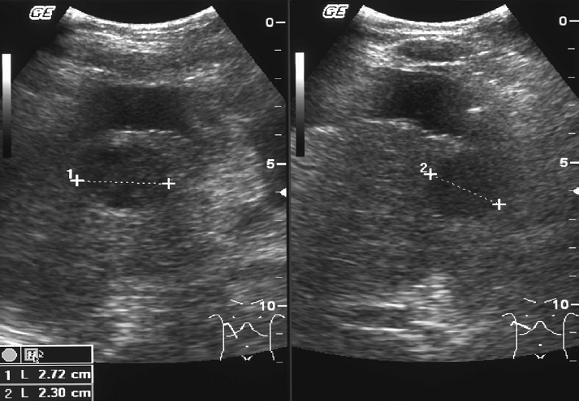
**Hypodense lesion (27 × 23 mm) in ultrasound**.

**Figure 2:**
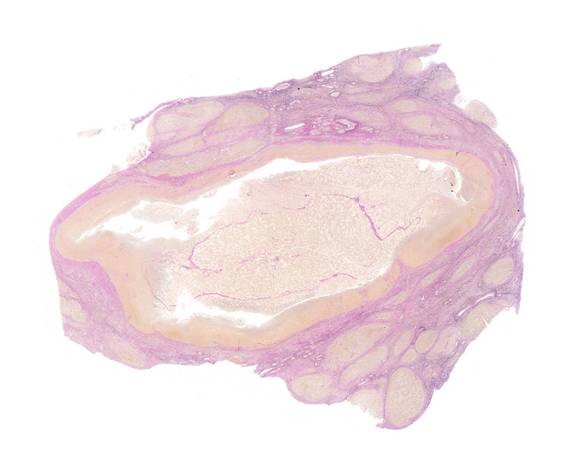
**Infarcted regenerative nodule in histopathology**.

One of the patients, a 54-year-old Caucasian male with viral hepatitis-induced liver cirrhosis, came to the emergency room due to acute respiratory insufficiency and hepatic encephalopathy grade IV. Chest x-ray revealed severe pneumonia. Except for the signs of liver cirrhosis the first abdominal CT scan did not show any pathological findings. However, 10 days later an abdominal ultrasound showed two new intrahepatic lesions (Figure 1). In spite of medical therapy the patient's liver function worsened with severe encephalopathy leading to death after one month. The autopsy revealed multiple fresh necrotic lesions in the liver. On histopathological examination two lesions (1.5 × 2.7 cm and 1.0 × 2.2 cm) were identified as infarcted regenerative nodules showing coagulative necrosis (Figure 2). The other lesions were described as necrotic lesions by chronic hepatitis C.

**Figure 3:**
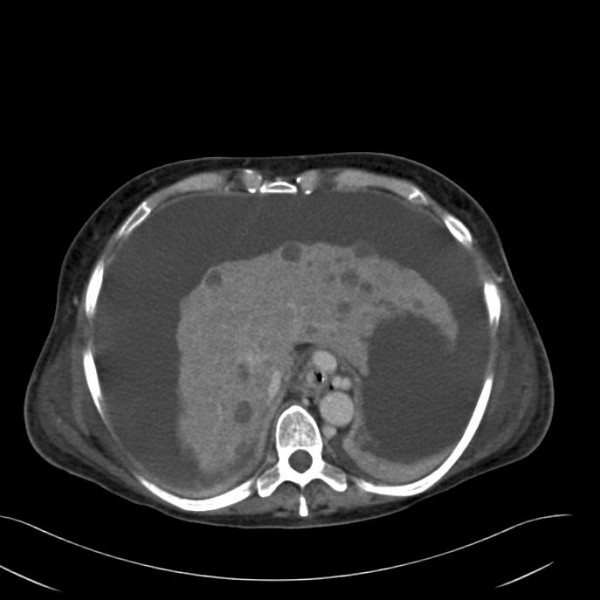
**CT scan with multiple new hypodense lesions**.

A 52-year-old Caucasian female patient had liver cirrhosis Child-Pugh C from Wilson's disease. She came to the hospital because of abdominal pain and ascites. During hospitalisation the patient was evaluated and listed for liver transplantation. Two weeks later oesophageal variceal bleeding and spontaneous bacterial peritonitis occurred, leading to a hepatorenal syndrome with increasing lactate. Therefore, the patient was transferred to the intensive care unit. An abdominal ultrasound and the following CT scan showed new hypodense lesions (Figure 3). One month after hospitalisation the patient died due to severe complications and multiple organ failure. Autopsy revealed multiple infarcted regenerative necrotic nodules.

The third patient, a 60-year-old Caucasian male with liver cirrhosis Child C, was admitted to the emergency room because of a collapse and hematemesis. On endoscopy, bleeding gastric varices were observed. Since bleeding was not stopped during endoscopy the patient received a TIPS (transjugular intrahepatic portosystemic shunt). In addition, spontaneous bacterial peritonitis was diagnosed. Two days after the TIPS was placed, an abdominal ultrasound was performed showing an isoechogenic lesion with a 2 cm diameter. During this time, the patient became more and more encephalopathic and finally he developed multiple organ failure and died one week later. Autopsy showed multiple widespread partially infected and infarcted regenerative nodules.

## Discussion

Recently, we have observed three cases with infarction of regenerative nodules, one patient with no variceal bleeding. Similar cases were reported as single case reports [1-4]. Kim *et al. *pointed out that especially in patients with a history of substantial gastrointestinal bleeding, infarcted regenerative nodules must be included in the list of differential diagnoses of focal liver lesions [3]. Microscopically, in our three cases, the degree of infarction was extensive. Hemorrhage and inflammatory changes were also observed around the infarcted regenerative nodules (Figure 2). In order to differentiate these lesions from malignant tumors, serial imaging seems to be necessary. Normally on unenhanced CT, typical regenerative nodules in cirrhosis are either not visible or appear with higher attenuation than adjacent parenchyma when they contain iron [3-6]. Such nodules are usually not visible on contrast-enhanced CT scans and appear isoattenuating to enhanced surrounding liver parenchyma. Similarly, only the siderotic regenerative nodules are visible as hypointense lesions on T2-weighted MR images [7-9]. In our patients, the lesions of infarcted regenerative nodules were depicted as different-appearing nodular lesions of low attenuation on unenhanced CT and as heterogeneous enhancement with regions of iso- and hypoattenuation relative to the surrounding liver on contrast-enhanced CT scans. In ultrasound, the image is like an abscess. In all of our patients the radiologists suggested liver abscesses. In MR imaging the appearance of such lesions is known to be different from that of regenerating nodules, showing high signal intensity on T2-weighted spin-echo MR images. Thus, these lesions can have different findings in cirrhotic livers and can be mistaken for a malignancy or even an abscess [10]. There are no data from autopsies and it is not known how often these infarcted regenerative nodules occur. Kang *et al. *reported on a vascular situation by regenerative nodules [1,2,5]. Hypotension can lead to a reduction of portal and arterial liver flow. Since variceal bleeding or septic shock can induce hypotension, as observed in our patients, we conclude that this could lead to infarction of nodules.

## Conclusion

In summary, our three patients showed an uncommon but important appearance of infarcted regenerative nodules in patients after decompensation of cirrhosis. It is important to note that these lesions can have a wide spectrum of imaging appearances with malignancies and abscesses included in the list of differential diagnoses. Serial imaging modalities will show rapidly appearing focal lesions. This feature of upgrowth can help in separation from tumors. The differential diagnosis of abscesses remains difficult and can often be made only at necropsy.

## Abbreviations

CT: computed tomography; MRI: magnetic resonance imaging; TIPS: transjugular intrahepatic portosystemic shunt

## Consent

Written informed consent could not be obtained from the patients for publication of this case series because the patients are now deceased and we were unable to contact their next-of-kin despite reasonable attempts. Every possible effort has been made to conceal the identity of the patients and we believe that reasonable families would not object to publication of this case series.

## Competing interests

The authors declare that they have no competing interests.

## Authors' contributions

SD, MB and GC analyzed and interpreted the patient data. RT performed the histological examination of the liver nodules. All authors read and approved the final manuscript.
